# Supplementary material for: Association between Statin Use and Gastric Cancer: A Nested Case-Control Study Using a National Health Screening Cohort in Korea
Source: Pharmaceuticals (Basel). 2021 Dec 8;14(12):1283. doi: 10.3390/ph14121283 (PMC8707102; doi:10.3390/ph14121283)
Supplement: Supplementary file 1 [file pharmaceuticals-14-01283-s001.zip › pharmaceuticals-1490653-Supplementary Tables 1-6 and Figures 1-2.pdf]

Supplementary Table S1 Crude and overlap propensity score weighted odd ratios of dates of any statin prescription for gastric cancer.

Characteristics	N of gastric cancer (exposure/total, %)	N of Control (exposure/total, %)	Odd ratios for gastric cancer (95% confidence interval)			
			Crude	*P*-value	Overlap weighted model †	*P*-value
Age < 65 years old (n= 20,780)						
< 180 days	3,759/4,156 (90.4%)	14,964/16,624 (90.0%)	1		1	
180 to 545 days	208/4,156 (5.0%)	872/16,624 (5.2%)	0.95 (0.81-1.11)	0.514	1.00 (0.88-1.14)	0.984
> 545 days	189/4,156 (4.5%)	788/16,624 (4.7%)	0.95 (0.81-1.12)	0.577	0.97 (0.85-1.11)	0.644
Age ≥ 65 years old (n= 23,210)						
< 180 days	3,961/4,642 (85.3%)	15,576/18,568 (83.9%)	1		1	
180 to 545 days	292/4,642 (6.3%)	1,353/18,568 (7.3%)	0.85 (0.74-0.97)	0.014*	0.81 (0.73-0.91)	<0.001*
> 545 days	389/4,642 (8.4%)	1,639/18,568 (8.8%)	0.93 (0.83-1.05)	0.243	0.94 (0.85-1.03)	0.186
Male (n= 32,355)						
< 180 days	5,740/6,471 (88.7%)	22,770/25,884 (88.0%)	1		1	
180 to 545 days	335/6,471 (5.2%)	1,440/25,884 (5.6%)	0.92 (0.82-1.04)	0.199	0.89 (0.80-0.98)	0.021*
> 545 days	396/6,471 (6.1%)	1,674/25,884 (6.5%)	0.94 (0.84-1.05)	0.271	0.93 (0.85-1.02)	0.127
Female (n= 11,635)						
< 180 days	1,980/2,327 (85.1%)	7,770/9,308 (83.5%)	1		1	
180 to 545 days	165/2,327 (7.1%)	785/9,308 (8.4%)	0.82 (0.69-0.98)	0.031*	0.87 (0.75-1.00)	0.051
> 545 days	182/2,327 (7.8%)	753/9,308 (8.1%)	0.95 (0.80-1.12)	0.540	1.02 (0.88-1.18)	0.801
Low income groups (n= 19,375)						
< 180 days	3,394/3,875 (87.6%)	13,604/15,500 (87.8%)	1		1	
180 to 545 days	239/3,875 (6.2%)	947/15,500 (6.1%)	1.01 (0.87-1.17)	0.878	1.01 (0.89-1.14)	0.918
> 545 days	242/3,875 (6.2%)	949/15,500 (6.1%)	1.02 (0.88-1.18)	0.768	1.10 (0.97-1.24)	0.137
High income groups (n= 24,615)						
< 180 days	4,326/4,923 (87.9%)	16,936/19,692 (86.0%)	1		1	
180 to 545 days	261/4,923 (5.3%)	1,278/19,692 (6.5%)	0.80 (0.70-0.92)	0.001*	0.79 (0.71-0.88)	<0.001*
> 545 days	336/4,923 (6.8%)	1,478/19,692 (7.5%)	0.89 (0.79-1.01)	0.064	0.87 (0.79-0.97)	0.010*
Urban residents (n= 18,070)						
< 180 days	3,132/3,614 (86.7%)	12,382/14,456 (85.7%)	1		1	
180 to 545 days	224/3,614 (6.2%)	975/14,456 (6.7%)	0.91 (0.78-1.06)	0.210	0.91 (0.81-1.03)	0.140
> 545 days	258/3,614 (7.1%)	1,099/14,456 (7.6%)	0.93 (0.81-1.07)	0.300	0.95 (0.85-1.07)	0.436
Rural residents (n= 25,920)						
< 180 days	4,588/5,184 (88.5%)	18,158/20,736 (87.6%)	1		1	
180 to 545 days	276/5,184 (5.3%)	1,250/20,736 (6.0%)	0.87 (0.76-1.00)	0.049*	0.86 (0.77-0.96)	0.006*
> 545 days	320/5,184 (6.2%)	1,328/20,736 (6.4%)	0.95 (0.84-1.08)	0.462	0.96 (0.86-1.07)	0.465
Underweight (n= 1,306)						
< 180 days	320/333 (96.1%)	909/973 (93.4%)	1		1	
180 to 545 days	6/333 (1.8%)	37/973 (3.8%)	0.46 (0.19-1.10)	0.082	0.63 (0.31-1.28)	0.200

> 545 days	7/333 (2.1%)	27/973 (2.8%)	0.74 (0.32-1.71)	0.476	0.93 (0.44-2.00)	0.861
Normal weight (n= 15,700)						
< 180 days	2,957/3,240 (91.3%)	11,262/12,460 (90.4%)	1		1	
180 to 545 days	139/3,240 (4.3%)	612/12,460 (4.9%)	0.87 (0.72-1.04)	0.132	0.87 (0.75-1.02)	0.086
> 545 days	144/3,240 (4.4%)	586/12,460 (4.7%)	0.94 (0.78-1.13)	0.487	0.91 (0.78-1.07)	0.268
Overweight (n= 11,986)						
< 180 days	1,970/2,285 (86.2%)	8,413/9,701 (86.7%)	1		1	
180 to 545 days	150/2,285 (6.6%)	600/9,701 (6.2%)	1.07 (0.89-1.29)	0.489	1.00 (0.86-1.16)	0.982
> 545 days	165/2,285 (7.2%)	688/9,701 (7.1%)	1.02 (0.86-1.22)	0.791	1.02 (0.88-1.18)	0.821
Obese (n= 14,998)						
< 180 days	2,473/2,940 (84.1%)	9,956/12,058 (82.6%)	1		1	
180 to 545 days	205/2,940 (7.0%)	976/12,058 (8.1%)	0.85 (0.72-0.99)	0.036*	0.82 (0.72-0.93)	0.002*
> 545 days	262/2,940 (8.9%)	1,126/12,058 (9.3%)	0.94 (0.81-1.08)	0.365	0.94 (0.84-1.06)	0.342
Non-smoker (n= 26,498)						
< 180 days	4,367/4,997 (87.4%)	18,615/21,501 (86.6%)	1		1	
180 to 545 days	286/4,997 (5.7%)	1,421/21,501 (6.6%)	0.86 (0.75-0.98)	0.022*	0.86 (0.78-0.96)	0.005*
> 545 days	344/4,997 (6.9%)	1,465/21,501 (6.8%)	1.00 (0.89-1.13)	0.988	1.05 (0.95-1.16)	0.343
Past and current smoker (n= 17,492)						
< 180 days	3,353/3,801 (88.2%)	11,925/13,691 (87.1%)	1		1	
180 to 545 days	214/3,801 (5.6%)	804/13,691 (5.9%)	0.95 (0.81-1.11)	0.490	0.91 (0.79-1.04)	0.148
> 545 days	234/3,801 (6.2%)	962/13,691 (7.0%)	0.87 (0.75-1.00)	0.055	0.84 (0.73-0.95)	0.006*
Alcohol consumption <1 time a week (n= 26,085)						
< 180 days	4,310/4,893 (88.1%)	18,354/21,192 (86.6%)	1		1	
180 to 545 days	280/4,893 (5.7%)	1,347/21,192 (6.4%)	0.89 (0.78-1.01)	0.072	0.90 (0.81-1.01)	0.062
> 545 days	303/4,893 (6.2%)	1,491/21,192 (7.0%)	0.87 (0.76-0.98)	0.027*	0.92 (0.83-1.02)	0.100
Alcohol consumption ≥1 time a week (n= 17,905)						
< 180 days	3,410/3,905 (87.3%)	12,186/14,000 (87.0%)	1		1	
180 to 545 days	220/3,905 (5.6%)	878/14,000 (6.3%)	0.90 (0.77-1.04)	0.156	0.84 (0.74-0.96)	0.008*
> 545 days	275/3,905 (7.0%)	936/14,000 (6.7%)	1.05 (0.91-1.21)	0.494	1.00 (0.89-1.14)	0.964
SBP < 140 mmHg and DBP < 90 mmHg (n= 31,460)						
< 180 days	5,563/6,300 (88.3%)	21,902/25,160 (87.1%)	1		1	
180 to 545 days	325/6,300 (5.2%)	1,514/25,160 (6.0%)	0.85 (0.75-0.96)	0.008*	0.86 (0.77-0.95)	0.002*
> 545 days	412/6,300 (6.5%)	1,744/25,160 (6.9%)	0.93 (0.83-1.04)	0.202	0.93 (0.85-1.03)	0.160
SBP ≥ 140 mmHg or DBP ≥ 90 mmHg (n= 12,530)						
< 180 days	2,157/2,498 (86.3%)	8,638/10,032 (86.1%)	1		1	
180 to 545 days	175/2,498 (7.0%)	711/10,032 (7.1%)	0.99 (0.83-1.17)	0.870	0.94 (0.81-1.08)	0.377
> 545 days	166/2,498 (6.6%)	683/10,032 (6.8%)	0.97 (0.82-1.16)	0.765	1.02 (0.88-1.18)	0.831
Fasting blood glucose < 100 mg/dL (n= 25,677)						
< 180 days	4,547/5,024 (90.5%)	18,532/20,653 (89.7%)	1		1	
180 to 545 days	236/5,024 (4.7%)	1,087/20,653 (5.3%)	0.88 (0.77-1.02)	0.097	0.94 (0.83-1.05)	0.278

> 545 days	241/5,024 (4.8%)	1,034/20,653 (5.0%)	0.95 (0.82-1.10)	0.484	0.97 (0.86-1.09)	0.588
Fasting blood glucose ≥ 100 mg/dL (n= 18,313)						
< 180 days	3,173/3,774 (84.1%)	12,008/14,539 (82.6%)	1		1	
180 to 545 days	264/3,774 (7.0%)	1,138/14,539 (7.8%)	0.88 (0.76-1.01)	0.067	0.84 (0.75-0.94)	0.003*
> 545 days	337/3,774 (8.9%)	1,393/14,539 (9.6%)	0.92 (0.81-1.04)	0.167	0.94 (0.85-1.05)	0.278
Total cholesterol < 200 mg/dL (n= 25,042)						
< 180 days	4,512/5,231 (86.3%)	16,995/19,811 (85.8%)	1		1	
180 to 545 days	278/5,231 (5.3%)	1,066/19,811 (5.4%)	0.98 (0.86-1.13)	0.797	0.92 (0.82-1.03)	0.139
> 545 days	441/5,231 (8.4%)	1,750/19,811 (8.8%)	0.95 (0.85-1.06)	0.351	0.96 (0.87-1.06)	0.459
Total cholesterol ≥ 200 mg/dL (n= 18,948)						
< 180 days	3,208/3,567 (89.9%)	13,545/15,381 (88.1%)	1		1	
180 to 545 days	222/3,567 (6.2%)	1,159/15,381 (7.5%)	0.81 (0.70-0.94)	0.005*	0.83 (0.74-0.94)	0.002*
> 545 days	137/3,567 (3.8%)	677/15,381 (4.4%)	0.85 (0.71-1.03)	0.100	0.86 (0.74-1.00)	0.050
CCI scores = 0 (n= 26,666)						
< 180 days	2,880/3,212 (89.7%)	20,905/23,454 (89.1%)	1		1	
180 to 545 days	166/3,212 (5.2%)	1,275/23,454 (5.4%)	0.95 (0.80-1.12)	0.506	0.96 (0.86-1.08)	0.539
> 545 days	166/3,212 (5.2%)	1,274/23,454 (5.4%)	0.95 (0.80-1.12)	0.511	0.95 (0.84-1.07)	0.380
CCI scores = 1 (n= 7,848)						
< 180 days	1,694/1,972 (85.9%)	4,883/5,876 (83.1%)	1		1	
180 to 545 days	111/1,972 (5.6%)	439/5,876 (7.5%)	0.73 (0.59-0.90)	0.004*	0.79 (0.66-0.96)	0.019*
> 545 days	167/1,972 (8.5%)	554/5,876 (9.4%)	0.87 (0.72-1.04)	0.130	1.01 (0.84-1.20)	0.940
CCI scores ≥ 2 (n= 9,476)						
< 180 days	3,146/3,614 (87.1%)	4,752/5,862 (81.1%)	1		1	
180 to 545 days	223/3,614 (6.2%)	511/5,862 (8.7%)	0.66 (0.56-0.78)	<0.001*	0.84 (0.71-0.98)	0.030*
> 545 days	245/3,614 (6.8%)	599/5,862 (10.2%)	0.62 (0.53-0.72)	<0.001*	0.90 (0.77-1.05)	0.181
Non dyslipidemia history (n= 25,900)						
< 180 days	5,397/5,514 (97.9%)	19,868/20,386 (97.5%)	1		1	
180 to 545 days	59/5,514 (1.1%)	276/20,386 (1.4%)	0.79 (0.59-1.04)	0.097	0.65 (0.52-0.80)	<0.001*
> 545 days	58/5,514 (1.1%)	242/20,386 (1.2%)	0.88 (0.66-1.18)	0.394	0.73 (0.59-0.92)	0.007*
Dyslipidemia history (n= 18,090)						
< 180 days	2,323/3,284 (70.7%)	10,672/14,806 (72.1%)	1		1	
180 to 545 days	441/3,284 (13.4%)	1,949/14,806 (13.2%)	1.04 (0.93-1.16)	0.500	0.93 (0.86-1.02)	0.124
> 545 days	520/3,284 (15.8%)	2,185/14,806 (14.8%)	1.09 (0.98-1.22)	0.098	0.96 (0.89-1.05)	0.410

Abbreviations: CCI, Charlson Comorbidity Index; SBP, Systolic blood pressure; DBP, Diastolic blood pressure

* Significance at $P < 0.05$

† Adjusted for age, sex, income, region of regidence, SBP, DBP, fasting blood glucose, total cholesterol, obesity, smoking, alcohol consumption, dyslipidemia history, and CCI scores.

Supplementary Table S2 Crude and overlap propensity score weighted odd ratios of dates of hydrophilic statin prescription for gastric cancer.

Characteristics	N of gastric cancer (exposure/total, %)	N of Control (exposure/total, %)	Odd ratios for gastric cancer (95% confidence interval)			
			Crude	*P*-value	Overlap weighted model †	*P*-value
Age < 65 years old (n= 20,780)						
< 180 days	4,091/4,156 (98.4%)	16,364/16,624 (98.4%)	1		1	
180 to 545 days	37/4,156 (0.9%)	152/16,624 (0.9%)	0.97 (0.68-1.40)	0.886	0.93 (0.70-1.23)	0.609
> 545 days	28/4,156 (0.7%)	108/16,624 (0.6%)	1.04 (0.68-1.57)	0.863	0.94 (0.68-1.31)	0.724
Age ≥ 65 years old (n= 23,210)						
< 180 days	4,511/4,642 (97.2%)	18,015/18,568 (97.0%)	1		1	
180 to 545 days	60/4,642 (1.3%)	304/18,568 (1.6%)	0.79 (0.60-1.04)	0.094	0.71 (0.57-0.88)	0.002*
> 545 days	71/4,642 (1.5%)	249/18,568 (1.3%)	1.14 (0.87-1.49)	0.338	1.06 (0.86-1.32)	0.592
Male (n= 32,355)						
< 180 days	6,332/6,471 (97.9%)	25,306/25,884 (97.8%)	1		1	
180 to 545 days	65/6,471 (1.0%)	312/25,884 (1.2%)	0.83 (0.64-1.09)	0.182	0.79 (0.65-0.97)	0.027*
> 545 days	74/6,471 (1.1%)	266/25,884 (1.0%)	1.11 (0.86-1.44)	0.423	1.04 (0.84-1.27)	0.737
Female (n= 11,635)						
< 180 days	2,270/2,327 (97.6%)	9,073/9,308 (97.5%)	1		1	
180 to 545 days	32/2,327 (1.4%)	144/9,308 (1.5%)	0.89 (0.60-1.31)	0.547	0.76 (0.56-1.02)	0.071
> 545 days	25/2,327 (1.1%)	91/9,308 (1.0%)	1.10 (0.70-1.71)	0.680	1.01 (0.70-1.45)	0.974
Low income groups (n= 19,375)						
< 180 days	3,792/3,875 (97.9%)	15,169/15,500 (97.9%)	1		1	
180 to 545 days	41/3,875 (1.1%)	196/15,500 (1.3%)	0.84 (0.60-1.17)	0.302	0.75 (0.57-0.97)	0.030*
> 545 days	42/3,875 (1.1%)	135/15,500 (0.9%)	1.24 (0.88-1.76)	0.218	1.26 (0.95-1.67)	0.108
High income groups (n= 24,615)						
< 180 days	4,810/4,923 (97.7%)	19,210/19,692 (97.6%)	1		1	
180 to 545 days	56/4,923 (1.1%)	260/19,692 (1.3%)	0.86 (0.64-1.15)	0.310	0.80 (0.64-1.00)	0.052
> 545 days	57/4,923 (1.2%)	222/19,692 (1.1%)	1.03 (0.77-1.37)	0.867	0.90 (0.72-1.14)	0.396
Urban residents (n= 18,070)						
< 180 days	3,513/3,614 (97.2%)	14,117/14,456 (97.7%)	1		1	
180 to 545 days	57/3,614 (1.6%)	185/14,456 (1.3%)	1.24 (0.92-1.67)	0.162	1.18 (0.92-1.51)	0.182
> 545 days	44/3,614 (1.2%)	154/14,456 (1.1%)	1.15 (0.82-1.61)	0.422	1.08 (0.83-1.42)	0.563
Rural residents (n= 25,920)						
< 180 days	5,089/5,184 (98.2%)	20,262/20,736 (97.7%)	1		1	
180 to 545 days	40/5,184 (0.8%)	271/20,736 (1.3%)	0.59 (0.42-0.82)	0.002*	0.52 (0.41-0.67)	<0.001*
> 545 days	55/5,184 (1.1%)	203/20,736 (1.0%)	1.08 (0.80-1.46)	0.620	0.99 (0.78-1.26)	0.958
Underweight (n= 1,306)						
< 180 days	331/333 (99.4%)	960/973 (98.7%)	1		1	
180 to 545 days	1/333 (0.3%)	9/973 (0.9%)	0.32 (0.04-2.55)	0.284	0.62 (0.13-3.06)	0.558

> 545 days	1/333 (0.3%)	4/973 (0.4%)	0.73 (0.08-6.51)	0.774	0.82 (0.14-4.87)	0.828
Normal weight (n= 15,700)						
< 180 days	3,186/3,240 (98.3%)	12,239/12,460 (98.2%)	1		1	
180 to 545 days	25/3,240 (0.8%)	136/12,460 (1.1%)	0.71 (0.46-1.08)	0.112	0.68 (0.49-0.94)	0.019*
> 545 days	29/3,240 (0.9%)	85/12,460 (0.7%)	1.31 (0.86-2.00)	0.210	1.13 (0.80-1.59)	0.477
Overweight (n= 11,986)						
< 180 days	2,231/2,285 (97.6%)	9,471/9,701 (97.6%)	1		1	
180 to 545 days	29/2,285 (1.3%)	111/9,701 (1.1%)	1.11 (0.74-1.67)	0.622	0.92 (0.67-1.25)	0.583
> 545 days	25/2,285 (1.1%)	119/9,701 (1.2%)	0.89 (0.58-1.38)	0.605	0.86 (0.62-1.20)	0.370
Obese (n= 14,998)						
< 180 days	2,854/2,940 (97.1%)	11,709/12,058 (97.1%)	1		1	
180 to 545 days	42/2,940 (1.4%)	200/12,058 (1.7%)	0.86 (0.62-1.20)	0.384	0.77 (0.59-1.00)	0.050*
> 545 days	44/2,940 (1.5%)	149/12,058 (1.2%)	1.21 (0.86-1.70)	0.267	1.09 (0.83-1.43)	0.531
Non-smoker (n= 26,498)						
< 180 days	4,888/4,997 (97.8%)	21,023/21,501 (97.8%)	1		1	
180 to 545 days	56/4,997 (1.1%)	277/21,501 (1.3%)	0.87 (0.65-1.16)	0.343	0.77 (0.62-0.95)	0.017*
> 545 days	53/4,997 (1.1%)	201/21,501 (0.9%)	1.13 (0.84-1.54)	0.418	1.07 (0.84-1.36)	0.575
Past and current smoker (n= 17,492)						
< 180 days	3,714/3,801 (97.7%)	13,356/13,691 (97.6%)	1		1	
180 to 545 days	41/3,801 (1.1%)	179/13,691 (1.3%)	0.82 (0.59-1.16)	0.265	0.79 (0.60-1.03)	0.083
> 545 days	46/3,801 (1.2%)	156/13,691 (1.1%)	1.06 (0.76-1.48)	0.728	0.98 (0.75-1.28)	0.878
Alcohol consumption <1 time a week (n= 26,085)						
< 180 days	4,792/4,893 (97.9%)	20,729/21,192 (97.8%)	1		1	
180 to 545 days	50/4,893 (1.0%)	253/21,192 (1.2%)	0.85 (0.63-1.16)	0.314	0.81 (0.65-1.02)	0.071
> 545 days	51/4,893 (1.0%)	210/21,192 (1.0%)	1.05 (0.77-1.43)	0.753	0.98 (0.77-1.24)	0.835
Alcohol consumption ≥1 time a week (n= 17,905)						
< 180 days	3,810/3,905 (97.6%)	13,650/14,000 (97.5%)	1		1	
180 to 545 days	47/3,905 (1.2%)	203/14,000 (1.5%)	0.83 (0.60-1.14)	0.251	0.73 (0.57-0.95)	0.017*
> 545 days	48/3,905 (1.2%)	147/14,000 (1.1%)	1.17 (0.84-1.62)	0.348	1.09 (0.83-1.44)	0.528
SBP < 140 mmHg and DBP < 90 mmHg (n= 31,460)						
< 180 days	6,160/6,300 (97.8%)	24,584/25,160 (97.7%)	1		1	
180 to 545 days	67/6,300 (1.1%)	312/25,160 (1.2%)	0.86 (0.66-1.12)	0.255	0.79 (0.64-0.97)	0.024*
> 545 days	73/6,300 (1.2%)	264/25,160 (1.0%)	1.10 (0.85-1.43)	0.459	1.05 (0.85-1.30)	0.646
SBP ≥ 140 mmHg or DBP ≥ 90 mmHg (n= 12,530)						
< 180 days	2,442/2,498 (97.8%)	9,795/10,032 (97.6%)	1		1	
180 to 545 days	30/2,498 (1.2%)	144/10,032 (1.4%)	0.84 (0.56-1.24)	0.374	0.76 (0.56-1.04)	0.083
> 545 days	26/2,498 (1.0%)	93/10,032 (0.9%)	1.12 (0.72-1.74)	0.607	1.00 (0.72-1.41)	0.984
Fasting blood glucose < 100 mg/dL (n= 25,677)						
< 180 days	4,940/5,024 (98.3%)	20,299/20,653 (98.3%)	1		1	

180 to 545 days	43/5,024 (0.9%)	213/20,653 (1.0%)	0.83 (0.60-1.15)	0.266	0.87 (0.68-1.12)	0.269
> 545 days	41/5,024 (0.8%)	141/20,653 (0.7%)	1.19 (0.84-1.69)	0.318	1.19 (0.90-1.57)	0.233
Fasting blood glucose ≥ 100 mg/dL (n= 18,313)						
< 180 days	3,662/3,774 (97.0%)	14,080/14,539 (96.8%)	1		1	
180 to 545 days	54/3,774 (1.4%)	243/14,539 (1.7%)	0.85 (0.63-1.15)	0.299	0.72 (0.57-0.91)	0.006*
> 545 days	58/3,774 (1.5%)	216/14,539 (1.5%)	1.03 (0.77-1.38)	0.831	0.94 (0.74-1.18)	0.586
Total cholesterol < 200 mg/dL (n= 25,042)						
< 180 days	5,090/5,231 (97.3%)	19,281/19,811 (97.3%)	1		1	
180 to 545 days	64/5,231 (1.2%)	258/19,811 (1.3%)	0.94 (0.71-1.24)	0.658	0.83 (0.67-1.04)	0.100
> 545 days	77/5,231 (1.5%)	272/19,811 (1.4%)	1.07 (0.83-1.38)	0.591	1.06 (0.86-1.29)	0.604
Total cholesterol ≥ 200 mg/dL (n= 18,948)						
< 180 days	3,512/3,567 (98.5%)	15,098/15,381 (98.2%)	1		1	
180 to 545 days	33/3,567 (0.9%)	198/15,381 (1.3%)	0.72 (0.49-1.04)	0.078	0.67 (0.51-0.89)	0.005*
> 545 days	22/3,567 (0.6%)	85/15,381 (0.6%)	1.11 (0.70-1.78)	0.656	0.82 (0.55-1.20)	0.305
CCI scores = 0 (n= 26,666)						
< 180 days	3,164/3,212 (98.5%)	23,088/23,454 (98.4%)	1		1	
180 to 545 days	28/3,212 (0.9%)	215/23,454 (0.9%)	0.95 (0.64-1.41)	0.804	0.94 (0.72-1.22)	0.646
> 545 days	20/3,212 (0.6%)	151/23,454 (0.6%)	0.97 (0.61-1.54)	0.888	0.96 (0.70-1.31)	0.785
CCI scores = 1 (n= 7,848)						
< 180 days	1,921/1,972 (97.4%)	5,669/5,876 (96.5%)	1		1	
180 to 545 days	19/1,972 (1.0%)	110/5,876 (1.9%)	0.51 (0.31-0.83)	0.007*	0.52 (0.35-0.77)	0.001*
> 545 days	32/1,972 (1.6%)	97/5,876 (1.7%)	0.97 (0.65-1.46)	0.896	1.10 (0.76-1.60)	0.599
CCI scores ≥ 2 (n= 9,476)						
< 180 days	3,517/3,614 (97.3%)	5,622/5,862 (95.9%)	1		1	
180 to 545 days	50/3,614 (1.4%)	131/5,862 (2.2%)	0.61 (0.44-0.85)	0.003*	0.82 (0.60-1.11)	0.204
> 545 days	47/3,614 (1.3%)	109/5,862 (1.9%)	0.69 (0.49-0.97)	0.034*	1.00 (0.72-1.38)	0.993
Non dyslipidemia history (n= 25,900)						
< 180 days	5,485/5,514 (99.5%)	20,286/20,386 (99.5%)	1		1	
180 to 545 days	13/5,514 (0.2%)	59/20,386 (0.3%)	0.81 (0.45-1.49)	0.505	0.60 (0.38-0.96)	0.034*
> 545 days	16/5,514 (0.3%)	41/20,386 (0.2%)	1.44 (0.81-2.57)	0.213	1.12 (0.71-1.76)	0.631
Dyslipidemia history (n= 18,090)						
< 180 days	3,117/3,284 (94.9%)	14,093/14,806 (95.2%)	1		1	
180 to 545 days	84/3,284 (2.6%)	397/14,806 (2.7%)	0.96 (0.75-1.21)	0.716	0.80 (0.67-0.96)	0.018*
> 545 days	83/3,284 (2.5%)	316/14,806 (2.1%)	1.19 (0.93-1.52)	0.169	0.98 (0.81-1.19)	0.855

Abbreviations: CCI, Charlson Comorbidity Index; SBP, Systolic blood pressure; DBP, Diastolic blood pressure

* Significance at $P < 0.05$

† Adjusted for age, sex, income, region of regidence, SBP, DBP, fasting blood glucose, total cholesterol, obesity, smoking, alcohol consumption, dyslipidemia history, and CCI scores.

Supplementary Table S3 Crude and overlap propensity score weighted odd ratios of dates of lipophilic statin prescription for gastric cancer.

Characteristics	N of gastric cancer	N of Control	Odd ratios for gastric cancer (95% confidence interval)			
	(exposure/total, %)	(exposure/total, %)	Crude	*P*-value	Overlap weighted model †	*P*-value
Age < 65 years old (n= 20,780)						
< 180 days	3,816/4,156 (91.8%)	15,197/16,624 (91.4%)	1		1	
180 to 545 days	192/4,156 (4.6%)	794/16,624 (4.8%)	0.96 (0.82-1.13)	0.648	1.02 (0.89-1.16)	0.787
> 545 days	148/4,156 (3.6%)	633/16,624 (3.8%)	0.93 (0.78-1.12)	0.443	0.97 (0.84-1.13)	0.719
Age ≥ 65 years old (n= 23,210)						
< 180 days	4,075/4,642 (87.8%)	16,049/18,568 (86.4%)	1		1	
180 to 545 days	272/4,642 (5.9%)	1,226/18,568 (6.6%)	0.87 (0.76-1.00)	0.052	0.85 (0.76-0.95)	0.006*
> 545 days	295/4,642 (6.4%)	1,293/18,568 (7.0%)	0.90 (0.79-1.02)	0.110	0.93 (0.84-1.04)	0.205
Male (n= 32,355)						
< 180 days	5,863/6,471 (90.6%)	23,274/25,884 (89.9%)	1		1	
180 to 545 days	312/6,471 (4.8%)	1,298/25,884 (5.0%)	0.95 (0.84-1.08)	0.469	0.92 (0.83-1.02)	0.119
> 545 days	296/6,471 (4.6%)	1,312/25,884 (5.1%)	0.90 (0.79-1.02)	0.095	0.91 (0.82-1.01)	0.084
Female (n= 11,635)						
< 180 days	2,028/2,327 (87.2%)	7,972/9,308 (85.6%)	1		1	
180 to 545 days	152/2,327 (6.5%)	722/9,308 (7.8%)	0.83 (0.69-0.99)	0.041*	0.90 (0.78-1.05)	0.182
> 545 days	147/2,327 (6.3%)	614/9,308 (6.6%)	0.94 (0.78-1.13)	0.524	1.05 (0.90-1.23)	0.512
Low income groups (n= 19,375)						
< 180 days	3,470/3,875 (89.5%)	13,894/15,500 (89.6%)	1		1	
180 to 545 days	222/3,875 (5.7%)	847/15,500 (5.5%)	1.05 (0.90-1.22)	0.535	1.06 (0.93-1.20)	0.383
> 545 days	183/3,875 (4.7%)	759/15,500 (4.9%)	0.97 (0.82-1.14)	0.677	1.05 (0.91-1.20)	0.510
High income groups (n= 24,615)						
< 180 days	4,421/4,923 (89.8%)	17,352/19,692 (88.1%)	1		1	
180 to 545 days	242/4,923 (4.9%)	1,173/19,692 (6.0%)	0.81 (0.70-0.93)	0.004*	0.82 (0.73-0.91)	0.001*
> 545 days	260/4,923 (5.3%)	1,167/19,692 (5.9%)	0.87 (0.76-1.00)	0.058	0.90 (0.80-1.01)	0.069
Urban residents (n= 18,070)						
< 180 days	3,219/3,614 (89.1%)	12,677/14,456 (87.7%)	1		1	
180 to 545 days	199/3,614 (5.5%)	898/14,456 (6.2%)	0.87 (0.74-1.02)	0.092	0.88 (0.77-1.00)	0.047*
> 545 days	196/3,614 (5.4%)	881/14,456 (6.1%)	0.88 (0.75-1.03)	0.104	0.92 (0.81-1.05)	0.238
Rural residents (n= 25,920)						
< 180 days	4,672/5,184 (90.1%)	18,569/20,736 (89.5%)	1		1	
180 to 545 days	265/5,184 (5.1%)	1,122/20,736 (5.4%)	0.94 (0.82-1.08)	0.368	0.95 (0.85-1.06)	0.351
> 545 days	247/5,184 (4.8%)	1,045/20,736 (5.0%)	0.94 (0.81-1.08)	0.390	0.98 (0.87-1.10)	0.766
Underweight (n= 1,306)						
< 180 days	322/333 (96.7%)	922/973 (94.8%)	1		1	
180 to 545 days	6/333 (1.8%)	29/973 (3.0%)	0.59 (0.24-1.44)	0.248	0.74 (0.35-1.57)	0.436

> 545 days	5/333 (1.5%)	22/973 (2.3%)	0.65 (0.24-1.73)	0.390	0.85 (0.35-2.03)	0.709
Normal weight (n= 15,700)						
< 180 days	3,007/3,240 (92.8%)	11,451/12,460 (91.9%)	1		1	
180 to 545 days	125/3,240 (3.9%)	542/12,460 (4.3%)	0.88 (0.72-1.07)	0.200	0.89 (0.75-1.04)	0.144
> 545 days	108/3,240 (3.3%)	467/12,460 (3.7%)	0.88 (0.71-1.09)	0.243	0.90 (0.75-1.07)	0.234
Overweight (n= 11,986)						
< 180 days	2,016/2,285 (88.2%)	8,623/9,701 (88.9%)	1		1	
180 to 545 days	142/2,285 (6.2%)	545/9,701 (5.6%)	1.11 (0.92-1.35)	0.266	1.09 (0.93-1.27)	0.306
> 545 days	127/2,285 (5.6%)	533/9,701 (5.5%)	1.02 (0.83-1.24)	0.852	1.04 (0.88-1.22)	0.668
Obese (n= 14,998)						
< 180 days	2,546/2,940 (86.6%)	10,250/12,058 (85.0%)	1		1	
180 to 545 days	191/2,940 (6.5%)	904/12,058 (7.5%)	0.85 (0.72-1.00)	0.050	0.84 (0.74-0.96)	0.008*
> 545 days	203/2,940 (6.9%)	904/12,058 (7.5%)	0.90 (0.77-1.06)	0.212	0.95 (0.83-1.08)	0.390
Non-smoker (n= 26,498)						
< 180 days	4,456/4,997 (89.2%)	19,029/21,501 (88.5%)	1		1	
180 to 545 days	273/4,997 (5.5%)	1,291/21,501 (6.0%)	0.90 (0.79-1.03)	0.138	0.92 (0.83-1.03)	0.141
> 545 days	268/4,997 (5.4%)	1,181/21,501 (5.5%)	0.97 (0.85-1.11)	0.652	1.05 (0.94-1.18)	0.356
Past and current smoker (n= 17,492)						
< 180 days	3,435/3,801 (90.4%)	12,217/13,691 (89.2%)	1		1	
180 to 545 days	191/3,801 (5.0%)	729/13,691 (5.3%)	0.93 (0.79-1.10)	0.398	0.90 (0.78-1.03)	0.139
> 545 days	175/3,801 (4.6%)	745/13,691 (5.4%)	0.84 (0.71-0.99)	0.037*	0.83 (0.72-0.95)	0.009*
Alcohol consumption <1 time a week (n= 26,085)						
< 180 days	4,400/4,893 (89.9%)	18,754/21,192 (88.5%)	1		1	
180 to 545 days	258/4,893 (5.3%)	1,242/21,192 (5.9%)	0.89 (0.77-1.02)	0.084	0.92 (0.82-1.02)	0.115
> 545 days	235/4,893 (4.8%)	1,196/21,192 (5.6%)	0.84 (0.73-0.97)	0.016*	0.92 (0.82-1.03)	0.166
Alcohol consumption ≥1 time a week (n= 17,905)						
< 180 days	3,491/3,905 (89.4%)	12,492/14,000 (89.2%)	1		1	
180 to 545 days	206/3,905 (5.3%)	778/14,000 (5.6%)	0.95 (0.81-1.11)	0.504	0.90 (0.79-1.04)	0.144
> 545 days	208/3,905 (5.3%)	730/14,000 (5.2%)	1.02 (0.87-1.19)	0.811	0.99 (0.87-1.14)	0.934
SBP < 140 mmHg and DBP < 90 mmHg (n= 31,460)						
< 180 days	5,687/6,300 (90.3%)	22,410/25,160 (89.1%)	1		1	
180 to 545 days	301/6,300 (4.8%)	1,369/25,160 (5.4%)	0.87 (0.76-0.98)	0.028*	0.90 (0.81-1.00)	0.040*
> 545 days	312/6,300 (5.0%)	1,381/25,160 (5.5%)	0.89 (0.78-1.01)	0.071	0.92 (0.82-1.02)	0.098
SBP ≥ 140 mmHg or DBP ≥ 90 mmHg (n= 12,530)						
< 180 days	2,204/2,498 (88.2%)	8,836/10,032 (88.1%)	1		1	
180 to 545 days	163/2,498 (6.5%)	651/10,032 (6.5%)	1.00 (0.84-1.20)	0.967	0.96 (0.83-1.11)	0.602
> 545 days	131/2,498 (5.2%)	545/10,032 (5.4%)	0.96 (0.79-1.17)	0.714	1.05 (0.90-1.24)	0.522
Fasting blood glucose < 100 mg/dL (n= 25,677)						
< 180 days	4,622/5,024 (92.0%)	18,847/20,653 (91.3%)	1		1	
180 to 545 days	219/5,024 (4.4%)	973/20,653 (4.7%)	0.92 (0.79-1.07)	0.263	0.97 (0.86-1.10)	0.660

> 545 days	183/5,024 (3.6%)	833/20,653 (4.0%)	0.90 (0.76-1.05)	0.186	0.91 (0.80-1.04)	0.174
Fasting blood glucose ≥ 100 mg/dL (n= 18,313)						
< 180 days	3,269/3,774 (86.6%)	12,399/14,539 (85.3%)	1		1	
180 to 545 days	245/3,774 (6.5%)	1,047/14,539 (7.2%)	0.89 (0.77-1.03)	0.105	0.87 (0.78-0.98)	0.026*
> 545 days	260/3,774 (6.9%)	1,093/14,539 (7.5%)	0.90 (0.78-1.04)	0.152	0.98 (0.87-1.10)	0.741
Total cholesterol < 200 mg/dL (n= 25,042)						
< 180 days	4,635/5,231 (88.6%)	17,441/19,811 (88.0%)	1		1	
180 to 545 days	255/5,231 (4.9%)	985/19,811 (5.0%)	0.97 (0.85-1.12)	0.717	0.92 (0.82-1.04)	0.195
> 545 days	341/5,231 (6.5%)	1,385/19,811 (7.0%)	0.93 (0.82-1.05)	0.223	0.96 (0.86-1.07)	0.440
Total cholesterol ≥ 200 mg/dL (n= 18,948)						
< 180 days	3,256/3,567 (91.3%)	13,805/15,381 (89.8%)	1		1	
180 to 545 days	209/3,567 (5.9%)	1,035/15,381 (6.7%)	0.86 (0.73-1.00)	0.047*	0.90 (0.79-1.01)	0.083
> 545 days	102/3,567 (2.9%)	541/15,381 (3.5%)	0.80 (0.64-0.99)	0.041*	0.87 (0.74-1.03)	0.101
CCI scores = 0 (n= 26,666)						
< 180 days	2,923/3,212 (91.0%)	21,224/23,454 (90.5%)	1		1	
180 to 545 days	154/3,212 (4.8%)	1,171/23,454 (5.0%)	0.95 (0.80-1.13)	0.600	0.98 (0.87-1.10)	0.746
> 545 days	135/3,212 (4.2%)	1,059/23,454 (4.5%)	0.93 (0.77-1.11)	0.409	0.93 (0.82-1.06)	0.268
CCI scores = 1 (n= 7,848)						
< 180 days	1,739/1,972 (88.2%)	5,060/5,876 (86.1%)	1		1	
180 to 545 days	105/1,972 (5.3%)	392/5,876 (6.7%)	0.78 (0.62-0.97)	0.028*	0.87 (0.71-1.06)	0.158
> 545 days	128/1,972 (6.5%)	424/5,876 (7.2%)	0.88 (0.72-1.08)	0.215	1.03 (0.85-1.25)	0.765
CCI scores ≥ 2 (n= 9,476)						
< 180 days	3,229/3,614 (89.3%)	4,962/5,862 (84.6%)	1		1	
180 to 545 days	205/3,614 (5.7%)	457/5,862 (7.8%)	0.69 (0.58-0.82)	<0.001*	0.87 (0.74-1.03)	0.117
> 545 days	180/3,614 (5.0%)	443/5,862 (7.6%)	0.62 (0.52-0.75)	<0.001*	0.90 (0.75-1.07)	0.216
Non dyslipidemia history (n= 25,900)						
< 180 days	5,425/5,514 (98.4%)	19,961/20,386 (97.9%)	1		1	
180 to 545 days	53/5,514 (1.0%)	235/20,386 (1.2%)	0.83 (0.62-1.12)	0.222	0.70 (0.55-0.88)	0.002*
> 545 days	36/5,514 (0.7%)	190/20,386 (0.9%)	0.70 (0.49-1.00)	0.048*	0.59 (0.45-0.77)	<0.001*
Dyslipidemia history (n= 18,090)						
< 180 days	2,466/3,284 (75.1%)	11,285/14,806 (76.2%)	1		1	
180 to 545 days	411/3,284 (12.5%)	1,785/14,806 (12.1%)	1.05 (0.94-1.18)	0.376	0.96 (0.88-1.06)	0.436
> 545 days	407/3,284 (12.4%)	1,736/14,806 (11.7%)	1.07 (0.95-1.21)	0.236	0.98 (0.90-1.08)	0.708

Abbreviations: CCI, Charlson Comorbidity Index; SBP, Systolic blood pressure; DBP, Diastolic blood pressure

* Significance at $P < 0.05$

† Adjusted for age, sex, income, region of regidence, SBP, DBP, fasting blood glucose, total cholesterol, obesity, smoking, alcohol consumption, dyslipidemia history, and CCI scores.

Supplementary Table S4 Crude and overlap propensity score weighted odd ratios of dates of statin prescription for mortality in gastric cancer participants.

Characteristics	Dead participants	Survived participants	Odd ratios for mortality (95% confidence interval)			
	(exposure/total, %)	(exposure/total, %)	Crude	*P*-value	Overlap weighted model †	*P*-value
Age < 65 years old (n= 4,156)						
< 180 days	690/737 (93.6%)	3,069/3,419 (89.8%)	1		1	
180 to 545 days	26/737 (3.5%)	182/3,419 (5.3%)	0.64 (0.42-0.97)	0.034*	1.07 (0.77-1.50)	0.684
> 545 days	21/737 (2.8%)	168/3,419 (4.9%)	0.56 (0.35-0.88)	0.013*	1.10 (0.76-1.60)	0.608
Age ≥ 65 years old (n= 4,642)						
< 180 days	1,414/1,572 (89.9%)	2,547/3,070 (83.0%)	1		1	
180 to 545 days	83/1,572 (5.3%)	209/3,070 (6.8%)	0.72 (0.55-0.93)	0.012*	1.17 (0.90-1.52)	0.236
> 545 days	75/1,572 (4.8%)	314/3,070 (10.2%)	0.43 (0.33-0.56)	<0.001*	0.80 (0.62-1.04)	0.091
Male (n= 6,471)						
< 180 days	1,617/1,761 (91.8%)	4,123/4,710 (87.5%)	1		1	
180 to 545 days	74/1,761 (4.2%)	261/4,710 (5.5%)	0.72 (0.55-0.94)	0.016*	0.97 (0.76-1.24)	0.828
> 545 days	70/1,761 (4.0%)	326/4,710 (6.9%)	0.55 (0.42-0.71)	<0.001*	0.86 (0.68-1.10)	0.231
Female (n= 2,327)						
< 180 days	487/548 (88.9%)	1,493/1,779 (83.9%)	1		1	
180 to 545 days	35/548 (6.4%)	130/1,779 (7.3%)	0.83 (0.56-1.22)	0.331	1.70 (1.20-2.42)	0.003*
> 545 days	26/548 (4.7%)	156/1,779 (8.8%)	0.51 (0.33-0.78)	0.002*	0.76 (0.52-1.11)	0.156
Low income groups (n= 3,875)						
< 180 days	1,043/1,150 (90.7%)	2,351/2,725 (86.3%)	1		1	
180 to 545 days	60/1,150 (5.2%)	179/2,725 (6.6%)	0.76 (0.56-1.02)	0.068	1.09 (0.82-1.45)	0.553
> 545 days	47/1,150 (4.1%)	195/2,725 (7.2%)	0.54 (0.39-0.75)	<0.001*	1.11 (0.80-1.54)	0.519
High income groups (n= 4,923)						
< 180 days	1,061/1,159 (91.5%)	3,265/3,764 (86.7%)	1		1	
180 to 545 days	49/1,159 (4.2%)	212/3,764 (5.6%)	0.71 (0.52-0.98)	0.036*	1.24 (0.93-1.64)	0.137
> 545 days	49/1,159 (4.2%)	287/3,764 (7.6%)	0.53 (0.39-0.72)	<0.001*	0.69 (0.53-0.90)	0.006*
Urban residents (n= 3,614)						
< 180 days	790/878 (90.0%)	2,342/2,736 (85.6%)	1		1	
180 to 545 days	45/878 (5.1%)	179/2,736 (6.5%)	0.75 (0.53-1.04)	0.087	1.03 (0.76-1.40)	0.850
> 545 days	43/878 (4.9%)	215/2,736 (7.9%)	0.59 (0.42-0.83)	0.002*	0.72 (0.53-0.96)	0.028*
Rural residents (n= 5,184)						
< 180 days	1,314/1,431 (91.8%)	3,274/3,753 (87.2%)	1		1	
180 to 545 days	64/1,431 (4.5%)	212/3,753 (5.6%)	0.75 (0.56-1.00)	0.052	1.26 (0.97-1.64)	0.084
> 545 days	53/1,431 (3.7%)	267/3,753 (7.1%)	0.49 (0.37-0.67)	<0.001*	0.95 (0.72-1.26)	0.722
Underweight (n= 333)						
< 180 days	164/168 (97.6%)	156/165 (94.5%)	1		1	
180 to 545 days	2/168 (1.2%)	4/165 (2.4%)	0.48 (0.09-2.63)	0.395	N/A	
> 545 days	2/168 (1.2%)	5/165 (3.0%)	0.38 (0.07-1.99)	0.253	0.62 (0.07-5.58)	0.671

Normal weight (n= 3,240)						
< 180 days	870/934 (93.1%)	2,087/2,306 (90.5%)	1		1	
180 to 545 days	35/934 (3.7%)	104/2,306 (4.5%)	0.81 (0.55-1.19)	0.283	1.54 (1.07-2.23)	0.020*
> 545 days	29/934 (3.1%)	115/2,306 (5.0%)	0.61 (0.40-0.92)	0.018*	0.94 (0.64-1.39)	0.765
Overweight (n= 2,285)						
< 180 days	507/560 (90.5%)	1,463/1,725 (84.8%)	1		1	
180 to 545 days	28/560 (5.0%)	122/1,725 (7.1%)	0.66 (0.43-1.01)	0.056	0.68 (0.47-1.00)	0.049*
> 545 days	25/560 (4.5%)	140/1,725 (8.1%)	0.52 (0.33-0.80)	0.003*	0.68 (0.47-1.00)	0.049*
Obese (n= 2,940)						
< 180 days	563/647 (87.0%)	1,910/2,293 (83.3%)	1		1	
180 to 545 days	44/647 (6.8%)	161/2,293 (7.0%)	0.93 (0.66-1.31)	0.669	1.26 (0.93-1.71)	0.142
> 545 days	40/647 (6.2%)	222/2,293 (9.7%)	0.61 (0.43-0.87)	0.006*	0.95 (0.70-1.28)	0.719
Non-smoker (n= 4,997)						
< 180 days	1,232/1,355 (90.9%)	3,135/3,642 (86.1%)	1		1	
180 to 545 days	68/1,355 (5.0%)	218/3,642 (6.0%)	0.79 (0.60-1.05)	0.106	1.31 (1.01-1.70)	0.045*
> 545 days	55/1,355 (4.1%)	289/3,642 (7.9%)	0.48 (0.36-0.65)	<0.001*	0.74 (0.57-0.97)	0.027*
Past and current smoker (n= 3,801)						
< 180 days	872/954 (91.4%)	2,481/2,847 (87.1%)	1		1	
180 to 545 days	41/954 (4.3%)	173/2,847 (6.1%)	0.67 (0.48-0.96)	0.027*	0.99 (0.73-1.35)	0.954
> 545 days	41/954 (4.3%)	193/2,847 (6.8%)	0.60 (0.43-0.85)	0.004*	1.03 (0.75-1.43)	0.848
Alcohol consumption <1 time a week (n= 4,893)						
< 180 days	1,376/1,508 (91.2%)	2,934/3,385 (86.7%)	1		1	
180 to 545 days	65/1,508 (4.3%)	215/3,385 (6.4%)	0.64 (0.49-0.86)	0.003*	1.04 (0.80-1.35)	0.786
> 545 days	67/1,508 (4.4%)	236/3,385 (7.0%)	0.61 (0.46-0.80)	<0.001*	0.98 (0.75-1.28)	0.894
Alcohol consumption ≥1 time a week (n= 3,905)						
< 180 days	728/801 (90.9%)	2,682/3,104 (86.4%)	1		1	
180 to 545 days	44/801 (5.5%)	176/3,104 (5.7%)	0.92 (0.66-1.29)	0.636	1.42 (1.05-1.93)	0.023*
> 545 days	29/801 (3.6%)	246/3,104 (7.9%)	0.43 (0.29-0.64)	<0.001*	0.66 (0.48-0.90)	0.010*
SBP < 140 mmHg and DBP < 90 mmHg (n= 6,300)						
< 180 days	1,411/1,522 (92.7%)	4,152/4,778 (86.9%)	1		1	
180 to 545 days	56/1,522 (3.7%)	269/4,778 (5.6%)	0.61 (0.46-0.82)	0.001*	1.09 (0.84-1.40)	0.511
> 545 days	55/1,522 (3.6%)	357/4,778 (7.5%)	0.45 (0.34-0.61)	<0.001*	0.72 (0.56-0.93)	0.011*
SBP ≥ 140 mmHg or DBP ≥ 90 mmHg (n= 2,498)						
< 180 days	693/787 (88.1%)	1,464/1,711 (85.6%)	1		1	
180 to 545 days	53/787 (6.7%)	122/1,711 (7.1%)	0.92 (0.66-1.28)	0.615	1.22 (0.88-1.70)	0.235
> 545 days	41/787 (5.2%)	125/1,711 (7.3%)	0.69 (0.48-1.00)	0.048*	1.07 (0.75-1.52)	0.713
Fasting blood glucose < 100 mg/dL (n= 5,024)						
< 180 days	1,232/1,324 (93.1%)	3,315/3,700 (89.6%)	1		1	
180 to 545 days	50/1,324 (3.8%)	186/3,700 (5.0%)	0.72 (0.53-1.00)	0.047*	1.11 (0.83-1.48)	0.490
> 545 days	42/1,324 (3.2%)	199/3,700 (5.4%)	0.57 (0.40-0.80)	0.001*	0.79 (0.58-1.09)	0.152

Fasting blood glucose ≥ 100 mg/dL (n= 3,774)						
< 180 days	872/985 (88.5%)	2,301/2,789 (82.5%)	1		1	
180 to 545 days	59/985 (6.0%)	205/2,789 (7.4%)	0.76 (0.56-1.03)	0.072	1.21 (0.92-1.59)	0.177
> 545 days	54/985 (5.5%)	283/2,789 (10.1%)	0.50 (0.37-0.68)	<0.001*	0.89 (0.68-1.17)	0.397
Total cholesterol < 200 mg/dL (n= 5,231)						
< 180 days	1,335/1,451 (92.0%)	3,177/3,780 (84.0%)	1		1	
180 to 545 days	55/1,451 (3.8%)	223/3,780 (5.9%)	0.59 (0.43-0.79)	0.001*	0.92 (0.70-1.21)	0.564
> 545 days	61/1,451 (4.2%)	380/3,780 (10.1%)	0.38 (0.29-0.50)	<0.001*	0.64 (0.49-0.83)	0.001*
Total cholesterol ≥ 200 mg/dL (n= 3,567)						
< 180 days	769/858 (89.6%)	2,439/2,709 (90.0%)	1		1	
180 to 545 days	54/858 (6.3%)	168/2,709 (6.2%)	1.02 (0.74-1.40)	0.905	1.51 (1.13-2.03)	0.006*
> 545 days	35/858 (4.1%)	102/2,709 (3.8%)	1.09 (0.74-1.61)	0.673	1.35 (0.95-1.92)	0.092
CCI scores = 0 (n= 3,212)						
< 180 days	225/245 (91.8%)	2,655/2,967 (89.5%)	1		1	
180 to 545 days	13/245 (5.3%)	153/2,967 (5.2%)	1.00 (0.56-1.79)	0.993	1.54 (1.06-2.24)	0.024*
> 545 days	7/245 (2.9%)	159/2,967 (5.4%)	0.52 (0.24-1.12)	0.095	0.82 (0.53-1.25)	0.350
CCI scores = 1 (n= 1,972)						
< 180 days	229/248 (92.3%)	1,465/1,724 (85.0%)	1		1	
180 to 545 days	11/248 (4.4%)	100/1,724 (5.8%)	0.70 (0.37-1.33)	0.280	1.44 (0.92-2.27)	0.110
> 545 days	8/248 (3.2%)	159/1,724 (9.2%)	0.32 (0.16-0.66)	0.002*	0.62 (0.39-0.98)	0.040*
CCI scores ≥ 2 (n= 3,614)						
< 180 days	1,650/1,816 (90.9%)	1,496/1,798 (83.2%)	1		1	
180 to 545 days	85/1,816 (4.7%)	138/1,798 (7.7%)	0.56 (0.42-0.74)	<0.001*	1.03 (0.77-1.39)	0.831
> 545 days	81/1,816 (4.5%)	164/1,798 (9.1%)	0.45 (0.34-0.59)	<0.001*	0.97 (0.72-1.31)	0.844
Non dyslipidemia history (n= 5,514)						
< 180 days	1,765/1,808 (97.6%)	3,632/3,706 (98.0%)	1		1	
180 to 545 days	25/1,808 (1.4%)	34/3,706 (0.9%)	1.51 (0.90-2.54)	0.118	1.16 (0.73-1.85)	0.521
> 545 days	18/1,808 (1.0%)	40/3,706 (1.1%)	0.93 (0.53-1.62)	0.788	0.79 (0.45-1.37)	0.399
Dyslipidemia history (n= 3,284)						
< 180 days	339/501 (67.7%)	1,984/2,783 (71.3%)	1		1	
180 to 545 days	84/501 (16.8%)	357/2,783 (12.8%)	1.38 (1.06-1.79)	0.018*	1.22 (1.00-1.48)	0.053
> 545 days	78/501 (15.6%)	442/2,783 (15.9%)	1.03 (0.79-1.35)	0.813	0.89 (0.73-1.09)	0.256
Only surgery (n= 7,259)						
< 180 days	1,247/1,377 (90.6%)	5,075/5,882 (86.3%)	1		1	
180 to 545 days	68/1,377 (4.9%)	365/5,882 (6.2%)	0.76 (0.58-0.99)	0.042*	1.03 (0.83-1.28)	0.777
> 545 days	62/1,377 (4.5%)	442/5,882 (7.5%)	0.57 (0.43-0.75)	<0.001*	0.85 (0.68-1.07)	0.164
Surgery+radiotherapy or chemotherapy (n= 1,539)						
< 180 days	857/932 (92.0%)	541/607 (89.1%)	1		1	
180 to 545 days	41/932 (4.4%)	26/607 (4.3%)	1.00 (0.60-1.65)	0.986	1.65 (0.97-2.81)	0.067
> 545 days	34/932 (3.6%)	40/607 (6.6%)	0.54 (0.34-0.86)	0.009*	0.84 (0.51-1.41)	0.518

Abbreviations: CCI, Charlson Comorbidity Index; SBP, Systolic blood pressure; DBP, Diastolic blood pressure.
* Significance at $P < 0.05$
† Adjusted for age, sex, income, region of regidence, SBP, DBP, fasting blood glucose, total cholesterol, obesity, smoking, alcohol consumption, dyslipidemia history, CCI scores and treatment type.

Supplementary Table S5 Crude and overlap propensity score weighted odd ratios of dates of hydrophilic prescription for mortality in gastric cancer participants.

Characteristics	Dead participants (exposure/total, %)	Survived participants (exposure/total, %)	Odd ratios for mortality (95% confidence interval) Crude	*P*-value	Overlap weighted model †	*P*-value
Age < 65 years old (n= 4,156)						
< 180 days	730/737 (99.1%)	3,361/3,419 (98.3%)	1		1	
180 to 545 days	6/737 (0.8%)	31/3,419 (0.9%)	0.89 (0.37-2.14)	0.797	0.97 (0.47-1.99)	0.933
> 545 days	1/737 (0.1%)	27/3,419 (0.8%)	0.17 (0.02-1.26)	0.083	0.12 (0.02-0.62)	0.012*
Age ≥ 65 years old (n= 4,642)						
< 180 days	1,544/1,572 (98.2%)	2,967/3,070 (96.6%)	1		1	
180 to 545 days	8/1,572 (0.5%)	52/3,070 (1.7%)	0.30 (0.14-0.62)	0.001*	0.28 (0.13-0.59)	0.001*
> 545 days	20/1,572 (1.3%)	51/3,070 (1.7%)	0.75 (0.45-1.27)	0.287	1.49 (0.88-2.54)	0.142
Male (n= 6,471)						
< 180 days	1,740/1,761 (98.8%)	4,592/4,710 (97.5%)	1		1	
180 to 545 days	8/1,761 (0.5%)	57/4,710 (1.2%)	0.37 (0.18-0.78)	0.009*	0.40 (0.22-0.76)	0.005*
> 545 days	13/1,761 (0.7%)	61/4,710 (1.3%)	0.56 (0.31-1.03)	0.061	0.96 (0.55-1.67)	0.888
Female (n= 2,327)						
< 180 days	534/548 (97.4%)	1,736/1,779 (97.6%)	1		1	
180 to 545 days	6/548 (1.1%)	26/1,779 (1.5%)	0.75 (0.31-1.83)	0.528	0.55 (0.25-1.22)	0.143
> 545 days	8/548 (1.5%)	17/1,779 (1.0%)	1.53 (0.66-3.57)	0.324	1.62 (0.76-3.41)	0.209
Low income groups (n= 3,875)						
< 180 days	1,134/1,150 (98.6%)	2,658/2,725 (97.5%)	1		1	
180 to 545 days	8/1,150 (0.7%)	33/2,725 (1.2%)	0.57 (0.26-1.23)	0.153	0.57 (0.29-1.10)	0.095
> 545 days	8/1,150 (0.7%)	34/2,725 (1.2%)	0.55 (0.25-1.20)	0.132	1.17 (0.55-2.48)	0.683
High income groups (n= 4,923)						
< 180 days	1,140/1,159 (98.4%)	3,670/3,764 (97.5%)	1		1	
180 to 545 days	6/1,159 (0.5%)	50/3,764 (1.3%)	0.39 (0.17-0.90)	0.028*	0.36 (0.17-0.76)	0.007*
> 545 days	13/1,159 (1.1%)	44/3,764 (1.2%)	0.95 (0.51-1.77)	0.875	1.15 (0.67-1.98)	0.601
Urban residents (n= 3,614)						
< 180 days	863/878 (98.3%)	2,650/2,736 (96.9%)	1		1	
180 to 545 days	8/878 (0.9%)	49/2,736 (1.8%)	0.50 (0.24-1.06)	0.072	0.32 (0.16-0.63)	0.001*
> 545 days	7/878 (0.8%)	37/2,736 (1.4%)	0.58 (0.26-1.31)	0.190	0.59 (0.31-1.16)	0.126
Rural residents (n= 5,184)						

< 180 days	1,411/1,431 (98.6%)	3,678/3,753 (98.0%)	1		1	
180 to 545 days	6/1,431 (0.4%)	34/3,753 (0.9%)	0.46 (0.19-1.10)	0.080	0.69 (0.34-1.40)	0.306
> 545 days	14/1,431 (1.0%)	41/3,753 (1.1%)	0.89 (0.48-1.64)	0.708	1.91 (1.04-3.52)	0.037*
Underweight (n= 333)						
< 180 days	168/168 (100.0%)	163/165 (98.8%)	1		1	
180 to 545 days	0/168 (0.0%)	1/165 (0.6%)	N/A		N/A	
> 545 days	0/168 (0.0%)	1/165 (0.6%)	N/A		N/A	
Normal weight (n= 3,240)						
< 180 days	922/934 (98.7%)	2,264/2,306 (98.2%)	1		1	
180 to 545 days	3/934 (0.3%)	22/2,306 (1.0%)	0.33 (0.10-1.12)	0.076	0.51 (0.19-1.34)	0.171
> 545 days	9/934 (1.0%)	20/2,306 (0.9%)	1.10 (0.50-2.44)	0.805	1.51 (0.72-3.16)	0.279
Overweight (n= 2,285)						
< 180 days	552/560 (98.6%)	1,679/1,725 (97.3%)	1		1	
180 to 545 days	3/560 (0.5%)	26/1,725 (1.5%)	0.35 (0.11-1.16)	0.087	0.19 (0.06-0.61)	0.006*
> 545 days	5/560 (0.9%)	20/1,725 (1.2%)	0.76 (0.28-2.04)	0.586	1.77 (0.70-4.48)	0.226
Obese (n= 2,940)						
< 180 days	632/647 (97.7%)	2,222/2,293 (96.9%)	1		1	
180 to 545 days	8/647 (1.2%)	34/2,293 (1.5%)	0.83 (0.38-1.80)	0.632	0.59 (0.31-1.13)	0.113
> 545 days	7/647 (1.1%)	37/2,293 (1.6%)	0.67 (0.30-1.50)	0.326	0.76 (0.38-1.52)	0.438
Non-smoker (n= 4,997)						
< 180 days	1,333/1,355 (98.4%)	3,555/3,642 (97.6%)	1		1	
180 to 545 days	9/1,355 (0.7%)	47/3,642 (1.3%)	0.51 (0.25-1.04)	0.066	0.44 (0.23-0.83)	0.012*
> 545 days	13/1,355 (1.0%)	40/3,642 (1.1%)	0.87 (0.46-1.63)	0.656	1.16 (0.66-2.03)	0.601
Past and current smoker (n= 3,801)						
< 180 days	941/954 (98.6%)	2,773/2,847 (97.4%)	1		1	
180 to 545 days	5/954 (0.5%)	36/2,847 (1.3%)	0.41 (0.16-1.05)	0.062	0.56 (0.26-1.20)	0.134
> 545 days	8/954 (0.8%)	38/2,847 (1.3%)	0.62 (0.29-1.33)	0.222	1.14 (0.55-2.33)	0.729
Alcohol consumption <1 time a week (n= 4,893)						
< 180 days	1,484/1,508 (98.4%)	3,308/3,385 (97.7%)	1		1	
180 to 545 days	8/1,508 (0.5%)	42/3,385 (1.2%)	0.42 (0.20-0.91)	0.027*	0.52 (0.27-0.99)	0.047*
> 545 days	16/1,508 (1.1%)	35/3,385 (1.0%)	1.02 (0.56-1.85)	0.951	1.64 (0.90-2.98)	0.108
Alcohol consumption ≥1 time a week (n= 3,905)						
< 180 days	790/801 (98.6%)	3,020/3,104 (97.3%)	1		1	
180 to 545 days	6/801 (0.7%)	41/3,104 (1.3%)	0.56 (0.24-1.32)	0.186	0.41 (0.19-0.88)	0.022*
> 545 days	5/801 (0.6%)	43/3,104 (1.4%)	0.44 (0.18-1.13)	0.087	0.67 (0.34-1.34)	0.258

SBP < 140 mmHg and DBP < 90 mmHg (n= 6,300)						
< 180 days	1,502/1,522 (98.7%)	4,658/4,778 (97.5%)	1		1	
180 to 545 days	7/1,522 (0.5%)	60/4,778 (1.3%)	0.36 (0.17-0.79)	0.011*	0.36 (0.19-0.67)	0.001*
> 545 days	13/1,522 (0.9%)	60/4,778 (1.3%)	0.67 (0.37-1.23)	0.196	0.96 (0.57-1.63)	0.882
SBP ≥ 140 mmHg or DBP ≥ 90 mmHg (n= 2,498)						
< 180 days	772/787 (98.1%)	1,670/1,711 (97.6%)	1		1	
180 to 545 days	7/787 (0.9%)	23/1,711 (1.3%)	0.66 (0.28-1.54)	0.336	0.76 (0.32-1.76)	0.516
> 545 days	8/787 (1.0%)	18/1,711 (1.1%)	0.96 (0.42-2.22)	0.927	1.77 (0.76-4.10)	0.185
Fasting blood glucose < 100 mg/dL (n= 5,024)						
< 180 days	1,307/1,324 (98.7%)	3,633/3,700 (98.2%)	1		1	
180 to 545 days	6/1,324 (0.5%)	37/3,700 (1.0%)	0.45 (0.19-1.07)	0.071	0.51 (0.24-1.09)	0.083
> 545 days	11/1,324 (0.8%)	30/3,700 (0.8%)	1.02 (0.51-2.04)	0.957	1.73 (0.85-3.55)	0.133
Fasting blood glucose ≥ 100 mg/dL (n= 3,774)						
< 180 days	967/985 (98.2%)	2,695/2,789 (96.6%)	1		1	
180 to 545 days	8/985 (0.8%)	46/2,789 (1.6%)	0.48 (0.23-1.03)	0.060	0.48 (0.25-0.91)	0.026*
> 545 days	10/985 (1.0%)	48/2,789 (1.7%)	0.58 (0.29-1.15)	0.120	0.94 (0.53-1.68)	0.832
Total cholesterol < 200 mg/dL (n= 5,231)						
< 180 days	1,432/1,451 (98.7%)	3,658/3,780 (96.8%)	1		1	
180 to 545 days	8/1,451 (0.6%)	56/3,780 (1.5%)	0.36 (0.17-0.77)	0.008*	0.38 (0.20-0.71)	0.003*
> 545 days	11/1,451 (0.8%)	66/3,780 (1.7%)	0.43 (0.22-0.81)	0.009*	0.98 (0.57-1.71)	0.949
Total cholesterol ≥ 200 mg/dL (n= 3,567)						
< 180 days	842/858 (98.1%)	2,670/2,709 (98.6%)	1		1	
180 to 545 days	6/858 (0.7%)	27/2,709 (1.0%)	0.70 (0.29-1.71)	0.440	0.68 (0.29-1.55)	0.354
> 545 days	10/858 (1.2%)	12/2,709 (0.4%)	2.64 (1.14-6.14)	0.024*	1.38 (0.66-2.90)	0.394
CCI scores = 0 (n= 3,212)						
< 180 days	245/245 (100.0%)	2,919/2,967 (98.4%)	1		1	
180 to 545 days	0/245 (0.0%)	28/2,967 (0.9%)	N/A		N/A	
> 545 days	0/245 (0.0%)	20/2,967 (0.7%)	N/A		N/A	
CCI scores = 1 (n= 1,972)						
< 180 days	244/248 (98.4%)	1,677/1,724 (97.3%)	1		1	
180 to 545 days	1/248 (0.4%)	18/1,724 (1.0%)	0.38 (0.05-2.87)	0.350	1.16 (0.25-5.34)	0.849
> 545 days	3/248 (1.2%)	29/1,724 (1.7%)	0.71 (0.21-2.35)	0.576	1.18 (0.50-2.75)	0.705
CCI scores ≥ 2 (n= 3,614)						
< 180 days	1785/1,816 (98.3%)	1,732/1,798 (96.3%)	1		1	
180 to 545 days	13/1,816 (0.7%)	37/1,798 (2.1%)	0.34 (0.18-0.64)	0.001*	0.54 (0.28-1.03)	0.062

> 545 days	18/1,816 (1.0%)	29/1,798 (1.6%)	0.60 (0.33-1.09)	0.093	1.53 (0.80-2.94)	0.202
Non dyslipidemia history (n= 5,514)						
< 180 days	1798/1,808 (99.4%)	3,687/3,706 (99.5%)	1		1	
180 to 545 days	5/1,808 (0.3%)	8/3,706 (0.2%)	1.28 (0.42-3.92)	0.664	0.59 (0.22-1.54)	0.279
> 545 days	5/1,808 (0.3%)	11/3,706 (0.3%)	0.93 (0.32-2.69)	0.896	1.09 (0.39-3.07)	0.866
Dyslipidemia history (n= 3,284)						
< 180 days	476/501 (95.0%)	2,641/2,783 (94.9%)	1		1	
180 to 545 days	9/501 (1.8%)	75/2,783 (2.7%)	0.67 (0.33-1.34)	0.255	0.44 (0.26-0.73)	0.002*
> 545 days	16/501 (3.2%)	67/2,783 (2.4%)	1.33 (0.76-2.31)	0.319	1.24 (0.80-1.91)	0.341
Only surgery (n= 7,259)						
< 180 days	1355/1,377 (98.4%)	5,733/5,882 (97.5%)	1		1	
180 to 545 days	7/1,377 (0.5%)	78/5,882 (1.3%)	0.38 (0.17-0.82)	0.014*	0.26 (0.14-0.52)	<0.001*
> 545 days	15/1,377 (1.1%)	71/5,882 (1.2%)	0.89 (0.51-1.57)	0.695	1.25 (0.78-2.02)	0.360
Surgery+radiotherapy or chemotherapy (n= 1,539)						
< 180 days	919/932 (98.6%)	595/607 (98.0%)	1		1	
180 to 545 days	7/932 (0.8%)	5/607 (0.8%)	0.91 (0.29-2.87)	0.867	1.15 (0.44-3.03)	0.775
> 545 days	6/932 (0.6%)	7/607 (1.2%)	0.55 (0.19-1.66)	0.292	1.03 (0.33-3.20)	0.958

Abbreviations: CCI, Charlson Comorbidity Index; SBP, Systolic blood pressure; DBP, Diastolic blood pressure;

* Significance at $P < 0.05$

† Adjusted for age, sex, income, region of regidence, SBP, DBP, fasting blood glucose, total cholesterol, obesity, smoking, alcohol consumption, dyslipidemia history, CCI scores and treatment type.

Supplementary Table S6 Crude and overlap propensity score weighted odd ratios of dates of lipophilic statin prescription for mortality in gastric cancer participants.

Characteristics	Dead participants (exposure/total, %)	Survived participants (exposure/total, %)	Odd ratios for mortality (95% confidence interval)			
			Crude	*P*-value	Overlap weighted model †	*P*-value
Age < 65 years old (n= 4,156)						
< 180 days	697/737 (94.6%)	3,119/3,419 (91.2%)	1		1	
180 to 545 days	22/737 (3.0%)	170/3,419 (5.0%)	0.58 (0.37-0.91)	0.018*	1.12 (0.78-1.60)	0.546
> 545 days	18/737 (2.4%)	130/3,419 (3.8%)	0.62 (0.38-1.02)	0.061	1.33 (0.89-1.99)	0.170
Age ≥ 65 years old (n= 4,642)						
< 180 days	1,440/1,572 (91.6%)	2,635/3,070 (85.8%)	1		1	
180 to 545 days	80/1,572 (5.1%)	192/3,070 (6.3%)	0.76 (0.58-1.00)	0.048*	1.24 (0.95-1.62)	0.109
> 545 days	52/1,572 (3.3%)	243/3,070 (7.9%)	0.39 (0.29-0.53)	<0.001*	0.72 (0.54-0.97)	0.030*
Male (n= 6,471)						
< 180 days	1,637/1,761 (93.0%)	4,226/4,710 (89.7%)	1		1	
180 to 545 days	71/1,761 (4.0%)	241/4,710 (5.1%)	0.76 (0.58-1.00)	0.048*	1.05 (0.82-1.35)	0.713
> 545 days	53/1,761 (3.0%)	243/4,710 (5.2%)	0.56 (0.42-0.76)	<0.001*	0.88 (0.67-1.15)	0.347
Female (n= 2,327)						
< 180 days	500/548 (91.2%)	1,528/1,779 (85.9%)	1		1	
180 to 545 days	31/548 (5.7%)	121/1,779 (6.8%)	0.78 (0.52-1.18)	0.239	1.79 (1.24-2.58)	0.002*
> 545 days	17/548 (3.1%)	130/1,779 (7.3%)	0.40 (0.24-0.67)	0.001*	0.63 (0.41-0.96)	0.034*
Low income groups (n= 3,875)						
< 180 days	1,058/1,150 (92.0%)	2,412/2,725 (88.5%)	1		1	
180 to 545 days	56/1,150 (4.9%)	166/2,725 (6.1%)	0.77 (0.56-1.05)	0.098	1.09 (0.81-1.47)	0.564
> 545 days	36/1,150 (3.1%)	147/2,725 (5.4%)	0.56 (0.39-0.81)	0.002*	1.18 (0.82-1.70)	0.368
High income groups (n= 4,923)						
< 180 days	1,079/1,159 (93.1%)	3,342/3,764 (88.8%)	1		1	
180 to 545 days	46/1,159 (4.0%)	196/3,764 (5.2%)	0.73 (0.52-1.01)	0.057	1.41 (1.06-1.89)	0.020*
> 545 days	34/1,159 (2.9%)	226/3,764 (6.0%)	0.47 (0.32-0.67)	<0.001*	0.61 (0.46-0.83)	0.001*
Urban residents (n= 3,614)						
< 180 days	803/878 (91.5%)	2,416/2,736 (88.3%)	1		1	
180 to 545 days	42/878 (4.8%)	157/2,736 (5.7%)	0.80 (0.57-1.14)	0.224	1.23 (0.89-1.71)	0.213
> 545 days	33/878 (3.8%)	163/2,736 (6.0%)	0.61 (0.42-0.89)	0.011*	0.85 (0.61-1.17)	0.319
Rural residents (n= 5,184)						

< 180 days	1,334/1,431 (93.2%)	3,338/3,753 (88.9%)	1		1	
180 to 545 days	60/1,431 (4.2%)	205/3,753 (5.5%)	0.73 (0.55-0.98)	0.038*	1.23 (0.94-1.61)	0.127
> 545 days	37/1,431 (2.6%)	210/3,753 (5.6%)	0.44 (0.31-0.63)	<0.001*	0.78 (0.57-1.07)	0.129
Underweight (n= 333)						
< 180 days	164/168 (97.6%)	158/165 (95.8%)	1		1	
180 to 545 days	2/168 (1.2%)	4/165 (2.4%)	0.48 (0.09-2.67)	0.403	N/A	
> 545 days	2/168 (1.2%)	3/165 (1.8%)	0.64 (0.11-3.90)	0.630	0.84 (0.09-8.19)	0.883
Normal weight (n= 3,240)						
< 180 days	882/934 (94.4%)	2,125/2,306 (92.2%)	1		1	
180 to 545 days	32/934 (3.4%)	93/2,306 (4.0%)	0.83 (0.55-1.25)	0.369	1.69 (1.15-2.50)	0.008*
> 545 days	20/934 (2.1%)	88/2,306 (3.8%)	0.55 (0.33-0.90)	0.016*	0.86 (0.55-1.36)	0.527
Overweight (n= 2,285)						
< 180 days	515/560 (92.0%)	1,501/1,725 (87.0%)	1		1	
180 to 545 days	27/560 (4.8%)	115/1,725 (6.7%)	0.68 (0.44-1.05)	0.084	0.78 (0.53-1.15)	0.209
> 545 days	18/560 (3.2%)	109/1,725 (6.3%)	0.48 (0.29-0.80)	0.005*	0.57 (0.37-0.88)	0.012*
Obese (n= 2,940)						
< 180 days	576/647 (89.0%)	1,970/2,293 (85.9%)	1		1	
180 to 545 days	41/647 (6.3%)	150/2,293 (6.5%)	0.93 (0.65-1.34)	0.712	1.25 (0.92-1.71)	0.158
> 545 days	30/647 (4.6%)	173/2,293 (7.5%)	0.59 (0.40-0.88)	0.010*	1.02 (0.73-1.42)	0.905
Non-smoker (n= 4,997)						
< 180 days	1,252/1,355 (92.4%)	3,204/3,642 (88.0%)	1		1	
180 to 545 days	64/1,355 (4.7%)	209/3,642 (5.7%)	0.78 (0.59-1.04)	0.097	1.33 (1.02-1.73)	0.037*
> 545 days	39/1,355 (2.9%)	229/3,642 (6.3%)	0.44 (0.31-0.62)	<0.001*	0.70 (0.52-0.94)	0.018*
Past and current smoker (n= 3,801)						
< 180 days	885/954 (92.8%)	2,550/2,847 (89.6%)	1		1	
180 to 545 days	38/954 (4.0%)	153/2,847 (5.4%)	0.72 (0.50-1.03)	0.071	1.08 (0.78-1.49)	0.659
> 545 days	31/954 (3.2%)	144/2,847 (5.1%)	0.62 (0.42-0.92)	0.018*	1.03 (0.72-1.47)	0.867
Alcohol consumption <1 time a week (n= 4,893)						
< 180 days	1,399/1,508 (92.8%)	3,001/3,385 (88.7%)	1		1	
180 to 545 days	61/1,508 (4.0%)	197/3,385 (5.8%)	0.66 (0.49-0.89)	0.006*	1.09 (0.83-1.43)	0.540
> 545 days	48/1,508 (3.2%)	187/3,385 (5.5%)	0.55 (0.40-0.76)	<0.001*	0.88 (0.65-1.19)	0.409
Alcohol consumption ≥1 time a week (n= 3,905)						
< 180 days	738/801 (92.1%)	2,753/3,104 (88.7%)	1		1	
180 to 545 days	41/801 (5.1%)	165/3,104 (5.3%)	0.93 (0.65-1.32)	0.672	1.54 (1.12-2.11)	0.008*
> 545 days	22/801 (2.7%)	186/3,104 (6.0%)	0.44 (0.28-0.69)	<0.001*	0.73 (0.51-1.04)	0.082

SBP < 140 mmHg and DBP < 90 mmHg (n= 6,300)						
< 180 days	1,431/1,522 (94.0%)	4,256/4,778 (89.1%)	1		1	
180 to 545 days	52/1,522 (3.4%)	249/4,778 (5.2%)	0.62 (0.46-0.84)	0.002*	1.18 (0.91-1.53)	0.218
> 545 days	39/1,522 (2.6%)	273/4,778 (5.7%)	0.42 (0.30-0.60)	<0.001*	0.70 (0.53-0.94)	0.017*
SBP ≥ 140 mmHg or DBP ≥ 90 mmHg (n= 2,498)						
< 180 days	706/787 (89.7%)	1,498/1,711 (87.6%)	1		1	
180 to 545 days	50/787 (6.4%)	113/1,711 (6.6%)	0.94 (0.67-1.33)	0.720	1.23 (0.88-1.73)	0.232
> 545 days	31/787 (3.9%)	100/1,711 (5.8%)	0.66 (0.44-0.99)	0.047*	0.99 (0.67-1.46)	0.965
Fasting blood glucose < 100 mg/dL (n= 5,024)						
< 180 days	1,247/1,324 (94.2%)	3,375/3,700 (91.2%)	1		1	
180 to 545 days	49/1,324 (3.7%)	170/3,700 (4.6%)	0.78 (0.56-1.08)	0.134	1.31 (0.96-1.77)	0.086
> 545 days	28/1,324 (2.1%)	155/3,700 (4.2%)	0.49 (0.33-0.73)	0.001*	0.65 (0.45-0.93)	0.019*
Fasting blood glucose ≥ 100 mg/dL (n= 3,774)						
< 180 days	890/985 (90.4%)	2,379/2,789 (85.3%)	1		1	
180 to 545 days	53/985 (5.4%)	192/2,789 (6.9%)	0.74 (0.54-1.01)	0.058	1.16 (0.88-1.54)	0.299
> 545 days	42/985 (4.3%)	218/2,789 (7.8%)	0.51 (0.37-0.72)	<0.001*	0.94 (0.70-1.27)	0.705
Total cholesterol < 200 mg/dL (n= 5,231)						
< 180 days	1,354/1,451 (93.3%)	3,281/3,780 (86.8%)	1		1	
180 to 545 days	50/1,451 (3.4%)	205/3,780 (5.4%)	0.59 (0.43-0.81)	0.001*	0.99 (0.75-1.31)	0.933
> 545 days	47/1,451 (3.2%)	294/3,780 (7.8%)	0.39 (0.28-0.53)	<0.001*	0.61 (0.46-0.81)	0.001*
Total cholesterol ≥ 200 mg/dL (n= 3,567)						
< 180 days	783/858 (91.3%)	2,473/2,709 (91.3%)	1		1	
180 to 545 days	52/858 (6.1%)	157/2,709 (5.8%)	1.05 (0.76-1.45)	0.785	1.59 (1.17-2.14)	0.003*
> 545 days	23/858 (2.7%)	79/2,709 (2.9%)	0.92 (0.57-1.47)	0.727	1.47 (0.98-2.21)	0.060
CCI scores = 0 (n= 3,212)						
< 180 days	225/245 (91.8%)	2,698/2,967 (90.9%)	1		1	
180 to 545 days	14/245 (5.7%)	140/2,967 (4.7%)	1.20 (0.68-2.11)	0.530	2.17 (1.48-3.19)	<0.001*
> 545 days	6/245 (2.4%)	129/2,967 (4.3%)	0.56 (0.24-1.28)	0.168	0.92 (0.57-1.49)	0.744
CCI scores = 1 (n= 1,972)						
< 180 days	233/248 (94.0%)	1,506/1,724 (87.4%)	1		1	
180 to 545 days	10/248 (4.0%)	95/1,724 (5.5%)	0.68 (0.35-1.32)	0.257	1.36 (0.86-2.16)	0.187
> 545 days	5/248 (2.0%)	123/1,724 (7.1%)	0.26 (0.11-0.65)	0.004*	0.51 (0.30-0.87)	0.014*
CCI scores ≥ 2 (n= 3,614)						
< 180 days	1,679/1,816 (92.5%)	1,550/1,798 (86.2%)	1		1	
180 to 545 days	78/1,816 (4.3%)	127/1,798 (7.1%)	0.57 (0.42-0.76)	<0.001*	1.03 (0.76-1.41)	0.842

> 545 days	59/1,816 (3.2%)	121/1,798 (6.7%)	0.45 (0.33-0.62)	<0.001*	0.94 (0.67-1.31)	0.697
Non dyslipidemia history (n= 5,514)						
< 180 days	1,775/1,808 (98.2%)	3,650/3,706 (98.5%)	1		1	
180 to 545 days	22/1,808 (1.2%)	31/3,706 (0.8%)	1.46 (0.84-2.53)	0.177	1.43 (0.86-2.37)	0.171
> 545 days	11/1,808 (0.6%)	25/3,706 (0.7%)	0.90 (0.44-1.84)	0.783	0.59 (0.29-1.22)	0.155
Dyslipidemia history (n= 3,284)						
< 180 days	362/501 (72.3%)	2,104/2,783 (75.6%)	1		1	
180 to 545 days	80/501 (16.0%)	331/2,783 (11.9%)	1.41 (1.07-1.84)	0.013*	1.25 (1.02-1.53)	0.032*
> 545 days	59/501 (11.8%)	348/2,783 (12.5%)	0.99 (0.73-1.33)	0.923	0.88 (0.71-1.09)	0.255
Only surgery (n= 7,259)						
< 180 days	1,268/1,377 (92.1%)	5,201/5,882 (88.4%)	1		1	
180 to 545 days	64/1,377 (4.6%)	341/5,882 (5.8%)	0.77 (0.59-1.01)	0.061	1.07 (0.86-1.32)	0.565
> 545 days	45/1,377 (3.3%)	340/5,882 (5.8%)	0.54 (0.40-0.75)	<0.001*	0.86 (0.67-1.10)	0.228
Surgery+radiotherapy or chemotherapy (n= 1,539)						
< 180 days	869/932 (93.2%)	553/607 (91.1%)	1		1	
180 to 545 days	38/932 (4.1%)	21/607 (3.5%)	1.15 (0.67-1.98)	0.611	1.98 (1.08-3.63)	0.027*
> 545 days	25/932 (2.7%)	33/607 (5.4%)	0.48 (0.28-0.82)	0.007*	0.73 (0.41-1.28)	0.267

Abbreviations: CCI, Charlson Comorbidity Index; SBP, Systolic blood pressure; DBP, Diastolic blood pressure;

* Significance at $P < 0.05$

† Adjusted for age, sex, income, region of regidence, SBP, DBP, fasting blood glucose, total cholesterol, obesity, smoking, alcohol consumption, dyslipidemia history, CCI scores and treatment type.

Supplementary Figure S1.

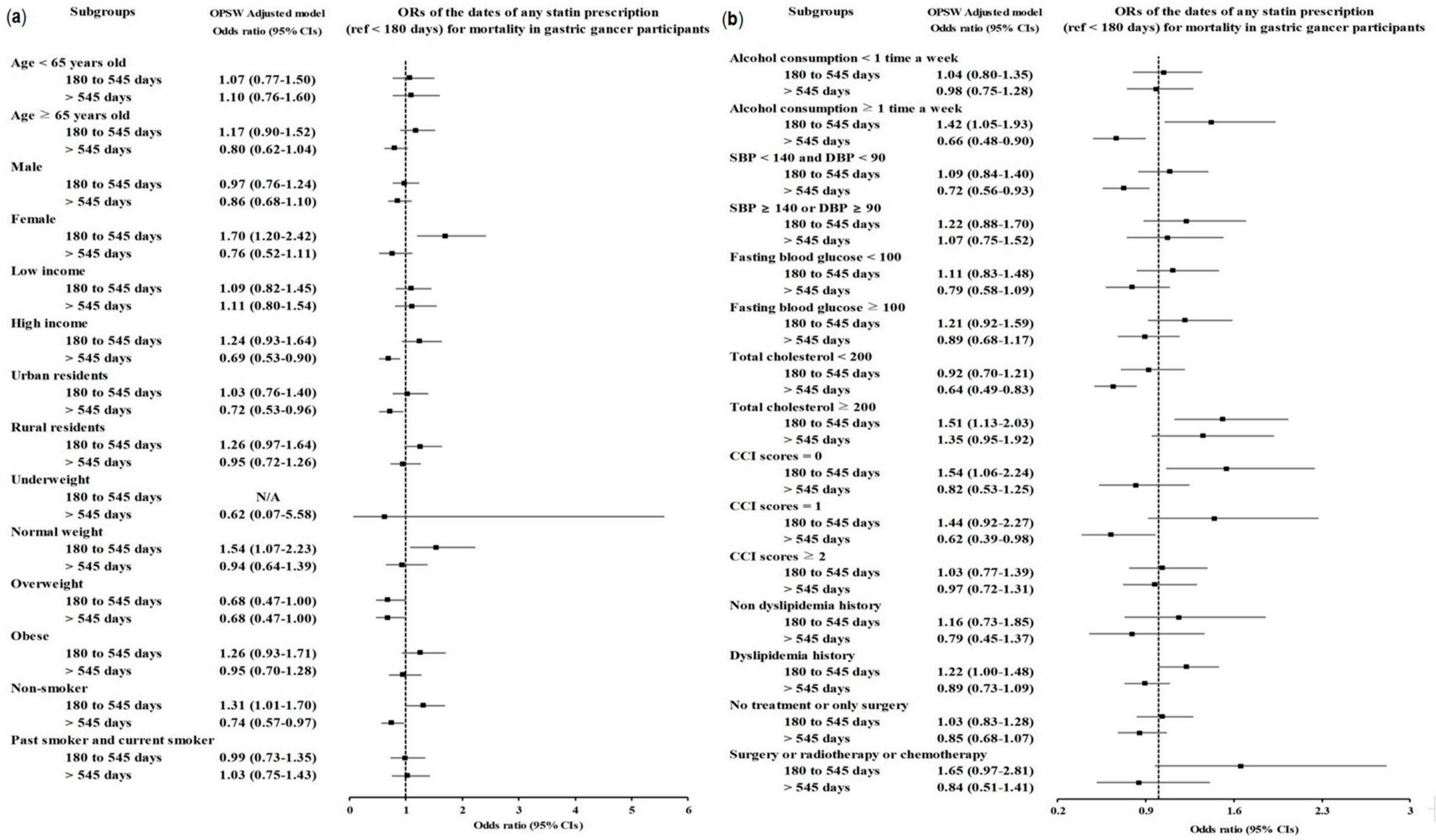

Supplementary Figure S1. Forest plots for multivariable conditional logistic regression depicting the overlap weighted odds ratios (95% confidence intervals) of previous use duration of any statin for overall mortality in the patients with incident gastric cancer according to comprehensive subgroup analyses including age, sex, income, region of residence, obesity, and smoking (**a**), alcohol consumption, systolic blood pressure, diastolic blood pressure, fasting blood glucose, total cholesterol, CCI scores, and dyslipidemia history (**b**). The reference period is <180 days. Full results of the crude and adjusted

overall weighted models are available in Supplementary Tables S4 (any statin).

Supplementary Figure S2.

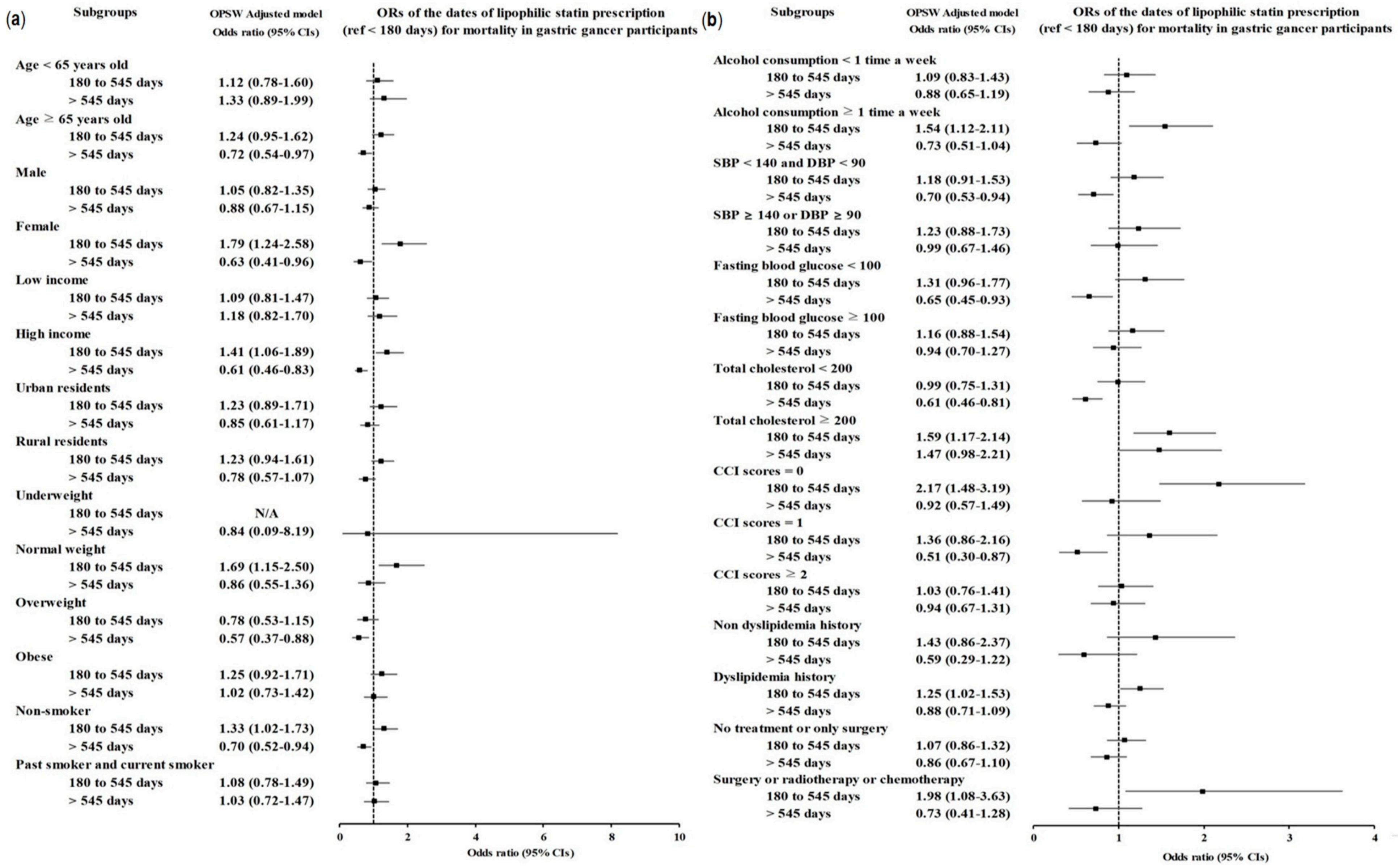

Supplementary Figure S2. Forest plots for multivariable conditional logistic regression depicting the overlap weighted odds ratios (95% confidence intervals) of previous use duration of lipophilic statin for overall mortality in the patients with incident gastric cancer according to comprehensive subgroup analyses including age, sex, income, region of residence, obesity, and smoking (**a**), alcohol consumption, systolic blood pressure, diastolic blood pressure, fasting blood glucose, total cholesterol, CCI scores, and dyslipidemia history (**b**). The reference period is <180 days. Full results of the crude and

adjusted overall weighted models are available in Supplementary Table S6 (lipophilic statin).
